# Clinical and radiation dose-volume factors related to pneumonitis after treatment with radiation and durvalumab in locally advanced non-small cell lung cancer

**DOI:** 10.1007/s10637-020-00917-2

**Published:** 2020-03-03

**Authors:** Hiroto Inoue, Akira Ono, Takanori Kawabata, Nobuaki Mamesaya, Takahisa Kawamura, Haruki Kobayashi, Shota Omori, Kazushige Wakuda, Hirotsugu Kenmotsu, Tateaki Naito, Haruyasu Murakami, Kazuaki Yasui, Hirofumi Ogawa, Tsuyoshi Onoe, Masahiro Endo, Hideyuki Harada, Toshiaki Takahashi

**Affiliations:** 1 Division of Thoracic Oncology, Shizuoka Cancer Center, 1007 Shimonagakubo, Nagaizumi, Sunto-gun, Shizuoka, 411-8777 Japan; 2 Clinical Research Center, Shizuoka Cancer Center, Shizuoka, Japan; 3 Division of Radiation Oncology, Shizuoka Cancer Center, Shizuoka, Japan; 4 Division of Diagnostic Radiology, Shizuoka Cancer Center, Shizuoka, Japan

**Keywords:** Durvalumab, Chemoradiotherapy, Pneumonitis, Risk factor, V20, Locally advanced non-small cell lung cancer

## Abstract

*Introduction* Durvalumab has been shown to confer a survival benefit after definitive chemoradiotherapy in the patients with locally advanced non-small cell lung cancer, but no studies have attempted to identify risk factors for pneumonitis after durvalumab therapy. The purpose of this study was to investigate associations between clinical and radiation dose-volume factors, and the severity of pneumonitis. *Methods* We retrospectively assessed the cases of 30 patients who had been started on durvalumab therapy between July 2018 and February 2019. In this study we evaluated the percentage of lung volume receiving radiation dose in excess of 20 Gy (V20) as radiation dose-volume factor. We compared V20 and some baseline factors between a grade 0 or 1 (Gr 0/1) pneumonitis group and a grade 2 or more (≥Gr 2) pneumonitis group, and we performed a logistic regression analysis to establish the associations between variables and ≥ Gr 2 pneumonitis. *Results* Pneumonitis had developed in 22 patients (73.3%): Gr 1/2/3–5 in 8 (26.7%)/14 (46.7%) /0 (0%), respectively. The difference in V20 between the Gr 0/1 group and Gr 2 group (median: 20.5% vs. 23.5%, *p* = 0.505) was not statistically significant, and thus V20 was not a risk factor for Gr 2 pneumonitis (odds ratio: 1.047, *p* = 0.303). None of the clinical factors, including sex, age, smoking history, presence of baseline pneumonitis, type of radiation therapy, location of lesion and facility, were risk factors. *Conclusions* Our study suggest that the severity of pneumonitis after durvalumab is unrelated to V20 or any of the clinical factors assessed in this study.

## Introduction

Definitive concurrent chemoradiotherapy (CRT) is a standard treatment for unresectable locally advanced non-small cell lung carcinoma (NSCLC). Preclinical studies have shown that radiation therapy synergistically enhances the antitumor effects of immunotherapy, such as with immune checkpoint inhibitors (ICIs), by increasing tumor infiltration and upregulating PD-L1 expression [1, 2]. In addition to its synergistic effect, previous radiotherapy has been reported to activate the antitumor immune response by causing cell death and releasing neoantigen from these dead tumor cells [3, 4]. Durvalumab is an ICI that selectively binds to PD-L1 with high affinity [5]. Durvalumab has been shown to confer a survival benefit after definitive CRT in patients with locally advanced NSCLC in the PACIFIC trial [6, 7].

CRT sometimes causes radiation pneumonitis. A meta-analysis reported that the incidence of radiation pneumonitis is 5%–50% rate, and that the mortality rate is 1%–2% [8], but it is unknown whether combining ICI therapy with CRT increases the risk of pneumonitis. The incidence of any grade (Gr) pneumonitis in the durvalumab arm of the PACIFIC trial was 33.9%, and the incidence of Gr 3/4 pneumonitis was 3.4%. but the incidences in the Japanese subgroup were higher: any Gr, 73.6% and Gr 3/4, 5.6%.

Lung V20, the percentage of lung volume receiving radiation dose in excess of 20 Gy, is the most common radiation dose-volume factor for radiation pneumonitis [9, 10]. Other risk factors for RP, including age, concurrent CRT regimen, location of the primary tumor lesion, and pre-existing lung disease, have also been reported [11–13], and female and never-smoker may be risk factors, but whether they are has been a matter of controversy [14, 15]. However, no studies have investigated whether any of these factors, including V20, are related to the development of pneumonitis after durvalumab administration following CRT, and there was no information about factors related to pneumonitis in the PACIFIC trial. We hypothesized that Lung V20 and other baseline clinical factors have an impact on the development of pneumonitis after durvalumab administration. The purpose of this study was to assess these factors for associations with the grade of pneumonitis.

## Methods

**Fig. 1:**
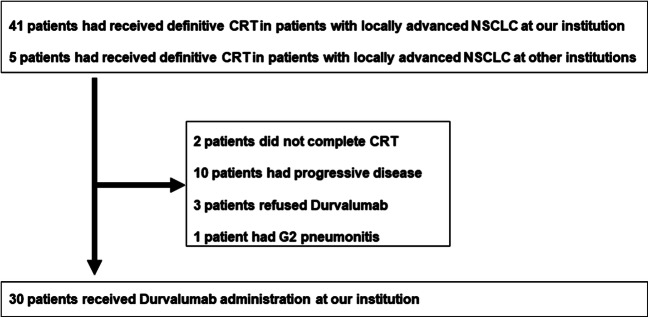
Flow diagram of the patients included in this study

Forty-one patients with stage III NSCLC received definitive CRT at our institution between April 2018 and December 2018, and 25 of these patients, with no progression after CRT, had been started on durvalumab between July 2018 and February 2019. Besides, another five patients received CRT at another institution and received durvalumab at our institution (Fig. 1). We retrospectively reviewed the data of these 30 patients. We decided whether CRT and durvalumab were indicated in each patient at a multidisciplinary conference. Durvalumab was administered in a dose of 10 mg/kg every 2 weeks until disease progression or intolerance, for up to 12 months. Twenty-one patients were treated with three-dimensional conformal radiation, and two patients were treated with intensity modulated radiation therapy (IMRT). Seven patients were treated with proton beam therapy (PBT) after approval was obtained at a multidisciplinary conference. We selected to use IMRT/PBT when the irradiation doses to normal tissue, such as lung and spinal cord, were likely to exceed acceptable range. The total radiation dose in all patients was 60 Gy or 64 Gy.

We compared V20 and baseline clinical factors between two groups, a Gr 0/1 pneumonitis group and ≥ Gr 2 pneumonitis group, and performed a univariate and multivariate analysis of risk factors for the pneumonitis. We diagnosed pneumonitis on the basis of the CT scan or X-ray evidence when a patient had a fever or cough, and, in the absence of symptoms, by chance at follow-up. Patients with an apparent pulmonary infection or heart failure were excluded. The grade of pneumonitis was evaluated according to the Common Terminology Criteria for Adverse Events (CTCAE) v.5.0. The patients with Gr 1 pneumonitis at baseline who did not experience an exacerbation after durvalumab administration were classified as Gr 0, and those with an exacerbation, as Gr 2. These criteria were consistent with those used in the Pacific trial.

Candidate variables for associations with Gr 2 pneumonitis included sex, age, smoking history, presence of Gr 1 pneumonitis at baseline, type of radiation therapy (X-ray or proton beam), location of the lesion (upper lobe or lower lobe), and facility where CRT was performed (our institution or other institution).

## Statistical analysis

Comparisons between categorical variables were performed using Fisher’s exact test, and comparisons between continuous variables (V20) were performed using Wilcoxon test. A multivariate logistic regression analysis was used to establish associations between these variables and ≥ Gr 2 pneumonitis. A *p* value of <0.05 was considered statistically significant in all analyses.

## Results

The patients’ characteristics are summarized in Table 1. Their median age was 68 years (range: 47–78 years). They included four patients who had received CRT for mediastinal lymph node (#3a, #4 L, #4R, #7) recurrence, and all four patients were in the upper lobe lesion group. The concurrent chemotherapy regimen was cisplatin + S-1 in 13 patients, carboplatin + paclitaxel in 7 patients, cisplatin + vinorelbine in 4 patients, daily carboplatin in 4 patients, and carboplatin + nab-paclitaxel in 7 patients. The PD-L1 tumor proportion score (TPS) with the 22C3 assay was calculated in 14 patients; 5 of them had a PD-L1 TPS <1%, and 4 of them had a PD-L1 TPS ≥50%.

**Table 1:** Characteristics of all patients and patients with Gr 0/1 and Gr 2 pneumonitis

		All	Gr 0/1	Gr 2	*p* value
	Number	30	16	14	
Age (years)	≥70	18	8	10	0.284
	<70	12	8	4	
Sex	Male	19	12	7	0.257
	Female	11	4	7	
Pathological type	Adeno	16	7	9	0.462
	Squamous	13	8	5	
	Unknown	1	1	0	
Clinical stage (UICC-8th edition)	IIIA	11	7	4	0.560
	IIIB	11	4	7	
	IIIC	4	2	2	
	LN re*	4	3	1	
Location	Upper lobe	20	12	8	0.442
	Lower lobe	10	4	6	
Smoking history	Yes	23	14	9	0.204
	No	7	2	5	
Baseline pneumonitis	Yes	6	4	2	0.657
	No	24	12	12	
Radiation	X-ray	23	13	10	0.204
	Proton beam	7	3	4	
V20	Median (range)	22 (5–34)	20.5 (5–34)	23.5 (10–32)	0.505

*LN re: lymph node recurrence

The median follow-up time was 7.7 months (range: 2.9–10.4 months). Six of the 30 patients had Gr 1 pneumonitis at baseline, and the pneumonitis exacerbated to Gr 2 in 2 of them. Of the 24 patients with no pneumonitis at baseline, 20 patients developed pneumonitis after durvalumab administration: Gr 1 in 8 patients and Gr 2 pneumonitis in 14 patients, including 2 patients with pneumonitis at baseline. No patients developed Gr 3–5 pneumonitis. Thus, pneumonitis developed in 22 patients (73.3%), and that of each grade was as follows: Gr 1/Gr 2/Gr 3–5 in 8 (26.7%)/14 (46.7%)/0 (0%), respectively. The pneumonitis developed at a median interval of 2.2 months (range: 0.6–3.4 months) after the completion of radiotherapy and a median of 1.4 months (range: 0.5–2.1 months) after the final dose of durvalumab. No pneumonitis developed beyond the radiation field.

Of the 14 patients with Gr 2 pneumonitis, 6 patients (27.3%) required prednisolone (PSL) therapy because their pneumonitis did not improve after durvalumab suspension, and all 6 patients recovered rapidly in response to PSL. In 10 patients (Gr 1: 3, Gr 2: 7), durvalumab could be re-administered after their pneumonitis was resolved. There were no exacerbations of pneumonitis during a median interval of 5.9 months (range: 1.1–7.5 months) after re-administration.

**Fig. 2:**
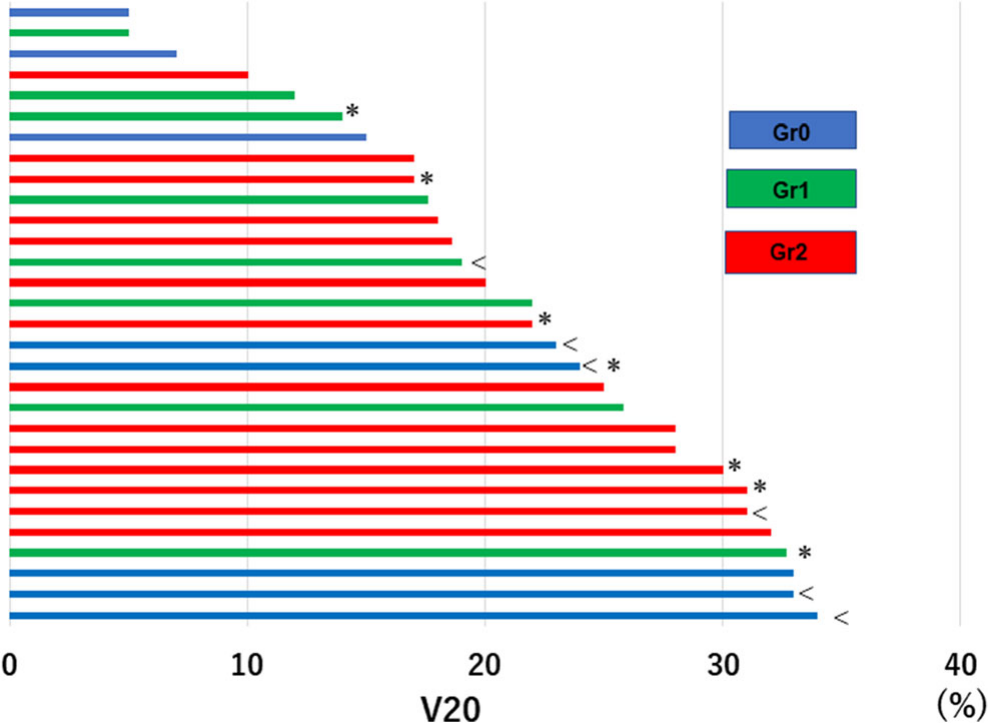
Relationship between grade of pneumonitis and V20. The cases are arranged in ascending order of their V20 values (plotted on the horizontal axis). Blue/green/red bars represent Gr 0/1/2 pneumonitis, respectively. “*” indicates treatment with proton beam therapy, and “<” indicates a case with Gr1 pneumonitis at baseline

The relation between the grade of pneumonitis and V20 is shown in Fig. 2. The results of the univariate analysis did not show a significant difference in V20 between the G 0/1 group and G2 group (median: 20.5% vs. 23.5%, *p* = 0.505, Wilcoxon test) (Table 1), and thus V20 could not be a risk factor for Gr 2 pneumonitis (odds ratio: 1.047, *p* = 0.303) (Table 2). Even when the cut-off value was set to 26% as in previous study [10], V20 could not be a risk factor (odds ratio: 2.25, *p* = 0.305). The results also showed that none of the other factors could be risk factors for Gr 2 pneumonitis (Table 2), and, thus, a multivariate analysis could not be performed.

**Table 2:** Univariate analysis of variables associated with Gr 2 pneumonitis

Variables		Odds ratio	*p* value
Age (years)	≥70 vs. <70	2.500	0.237
Sex	Male vs. Female	0.333	0.163
Pathological type	Adeno vs. Squamous	0.486	0.343
Location	Lower lobe vs. Upper lobe	2250	0.305
Smoking history	Yes vs. No	0.257	0.148
Baseline pneumonitis	Yes vs. No	0.500	0.470
Radiation	Proton beam vs. X-ray	3.889	0.148
Facility	Our institution vs. others	0.524	0.517
V20	≥26 vs. <26	2.25	0.305
V20 (continuous)		1.047	0.303

## Discussion

We report the relation of V20 and some baseline clinical factors to the grade of pneumonitis after durvalumab administration following CRT. Although radiation pneumonitis has reported to be associated with V20 and some clinical factors in the previous studies, the results of our study did not show a statistically significant association between any of the factors, including V20, and pneumonitis after durvalumab administration. To our knowledge, this is the first study to attempt to identify risk factors for pneumonitis after durvalumab administration.

The incidence of pneumonitis in this study was almost the same as in the Japanese subgroup of the PACIFIC trial. However, none of the patients in our study developed severe pneumonitis (Gr 3–5), and only a few patients developed severe pneumonitis in the PACIFIC trial. Since the Japanese clinical guideline for lung cancer state that patients with a V20 ≥ 35% are highly likely to develop radiation pneumonitis, we avoid irradiating such patients in consultation with radiation oncologists. This patient selection perhaps prevented the development of severe pneumonitis. Interestingly, a smaller percentage of patients with pneumonitis required PSL administration in our study (27.3%) than in the PACIFIC trial (62.1%), because the protocol of the PACIFIC trial recommended that all patients with Gr 2 pneumonitis be started on systemic steroid therapy promptly [6]. On the other hand, 7 (50%) of the 14 patients with Gr 2 pneumonitis in our study were cured by durvalumab suspension alone. This may be explained by the fact that we monitored the course of pneumonitis without PSL therapy for one to two weeks in accordance with the strategy for radiation pneumonitis [16]. Another reason might be that no pneumonitis developed beyond the radiation field because of proper radiation treatment planning. Not all patients with Gr 2 pneumonitis may require PSL therapy, but we could not identify the clinical differences between the group of patients who needed PSL and the group that did not, because the number of subjects was small.

The results of this study did not show any statistically significant associations between V20 and the grade of pneumonitis after durvalumab administration. As stated above, there were no patients with a V20 ≥ 35% in this study. Tsujino K et al. [10] reported finding that the 6-month cumulative incidence of Gr 2 radiation pneumonitis increased with V20 even when V20 was less than 35%. The pneumonitis after durvalumab administration is induced by the ICI as well as by the radiation, so factors other than dosimetric factors such as V20 may be more important. Radiation therapy induces tumor cell death, which leads to an increase in tumor-infiltrating lymphocytes (TILs) in the microenvironment and to upregulation of PD-L1 on the surface of the tumor [2]. These factors may be responsible for the differences in immune-related adverse events across patients or tumor types [17]. Similarly, biomarkers such as TILs and PD-L1 are likely to influence the severity of the pneumonitis after durvalumab administration.

The pneumonitis that develops after durvalumab is different from radiation pneumonitis in terms of radiological features. Although it is difficult to distinguish between the two forms of pneumonitis, ICI-related pneumonitis has been reported to exhibit some specific radiological features, such as ground glass opacities (GGOs), a cryptogenic organizing pneumonia-like appearance, and interstitial pneumonia pattern [18]. Baba T et al. [19] reported that GGOs around the tumor (peritumoral infiltration) were a characteristic finding in pneumonitis induced by nivolumab.

ICIs, including durvalumab, have caused pneumonitis in the rate of 2.7%–5% in some recent reports [20–22]. Few studies have identified baseline clinical factors associated with the ICI-induced pneumonitis. Naidoo J et al. [22] reported significant associations between both current/former smokers and underlying lung conditions and the worsening of pneumonitis by ICI monotherapy or ICI combination therapy. However, smoking history and baseline pneumonitis were not found to be risk factors in our study. Unfortunately, we were unable to identify any clinical factors associated with the pneumonitis after durvalumab administration following CRT. The involvement of both radiation and ICIs may complicate the detection of the risk factors for this pneumonitis.

This study had two limitations. First, the subjects were a small number of patients at a single institution. Second, the follow-up period was too short to draw a conclusion. Although all of the cases of pneumonitis in this study developed within 3 months after the final durvalumab dose, pneumonitis is likely to develop after follow-up time. Further study with a longer follow-up time will be needed to confirm our results.

In conclusion, the results of our study suggest that the severity of pneumonitis after durvalumab administration are unrelated to V20 or any other clinical factors that we assessed in this study. Further investigations are warranted, for finding out other factors that affect developing the pneumonitis.
